# Dimma: Semi-Supervised Low-Light Image Enhancement with Adaptive Dimming

**DOI:** 10.3390/e26090726

**Published:** 2024-08-26

**Authors:** Wojciech Kozłowski, Michał Szachniewicz, Michał Stypułkowski, Maciej Zięba

**Affiliations:** 1 Faculty of Information and Communication Technology, Wrocław University of Science and Technology, 50-370 Wrocław, Poland; 2 Faculty of Mathematics and Computer Science, University of Wrocław, 50-384 Wrocław, Poland; michal.stypulkowski96@gmail.com; 3 Tooploox Ltd., 53-601 Wrocław, Poland

**Keywords:** computer vision, image enhancement, low-light image enhancement, semi-supervised learning

## Abstract

Enhancing low-light images with natural colors poses a challenge due to camera processing variations and limited access to ground-truth lighting conditions. To address this, we propose Dimma, a semi-supervised approach that aligns with any camera using a small set of image pairs captured under extreme lighting conditions. Our method employs a convolutional mixture density network to replicate camera-specific noise present in dark images. We enhance results further by introducing a conditional UNet architecture based on user-provided lightness values. Trained on just a few real image pairs, Dimma achieves competitive results compared to fully supervised state-of-the-art methods trained on large datasets.

## 1. Introduction

The low-light image enhancement task aims to replicate the appearance of a photo taken with a longer exposure by reducing noise, increasing brightness, and preserving natural colors. Many research papers [1,2,3,4,5] have reported promising results on paired datasets containing well-lit and dark images. However, the effectiveness of models trained on one dataset may not generalize well to images from different cameras. Variations in how individual cameras process images pose a challenge to achieving consistent and reliable enhancement across diverse camera models. Creating a comprehensive set of paired images for numerous devices is not only challenging but also expensive, as it involves taking two images of the same scene with different exposure times, requiring static scenes and precision in capturing the photos in order to align the images. This slow process results in datasets with limited and very similar pairs, often taken by the same camera in the same location, such as the LOL dataset [1] with only 480 training pairs.

On the other hand, unsupervised methods [6,7] do not require a paired dataset in the training process; therefore, they are not biased towards limited training data and can generalize better. However, lacking defined ground-truth in the training process often leads to lower-quality results obtained by unsupervised methods.

**Figure 1:**
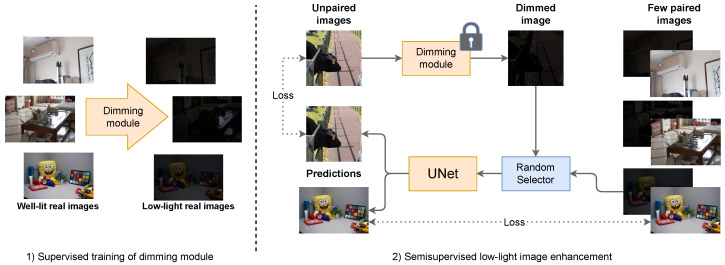
High-level diagram of our proposed method. (1) We train the dimming module to mimic color distortion made by a specific camera. (2) We employ this dimming module and the same few pairs to train a UNet [8] architecture in a semi-supervised fashion. With fixed probability, the random selector takes either a fake or a real dark image and learns to predict its light version. In the second phase, the parameters of the dimming module are frozen.

To fill this gap, we propose a semi-supervised approach (Figure 1) that can leverage a small set of real paired images to construct a dimming module capable of replicating the camera-specific dimming process. In this way, we reduce the time needed to collect comprehensive datasets for different cameras. By utilizing a mixture density network and illumination statistics extracted from the real image pairs, the dimming module can effectively darken an image and mimic the color distortion associated with very dark images from a selected source.

Next, we create dimmed versions of images from various computer vision datasets using the constructed dimming module and train a UNet [8] model to restore their original light conditions. Our UNet architecture incorporates a brightness level conditioning to represent light differences and predict the contrast between dark and light images, rather than directly predicting the light values themselves. This approach allows the model to learn conditioned image light enhancement, providing flexibility and adaptability to various lighting conditions.

Our proposed approach offers a practical and cost-effective solution for training image enhancement models using only few paired examples. The utilization of a dimming module based on a few real paired images enables the model to mimic the context-specific dimming process accurately. Furthermore, the ability to adjust the brightening factor provides additional control over the image enhancement process, which is not feasible when relying solely on paired datasets.

Through extensive experimentation and evaluation, we demonstrate the effectiveness of our approach in enhancing low-light images while preserving natural colors. By addressing the challenges posed by camera variations, the limited availability of paired datasets, and the control over the brightening factor, our research contributes to advancing the field of low-light image enhancement and provides a practical and adaptable solution for real-world applications.

In summary, our contributions can be outlined as follows:

- We introduce a dimming module based on a mixture density network [9] and illumination statistics calculated from a small set of ground-truth image pairs. This approach replicates the color distortion and darkening process of dark images and enables semi-supervised learning of the brightening module using as few as three image pairs.
- We propose to use UNet [8] conditioned on light differences to predict the residuals between dark and light images rather than the light values themselves.
- We introduce two datasets: the FewShow-Dark (FS-Dark) dataset containing a few real image pairs captured by mobile phone cameras and the MixHQ dataset, which comprises high-quality images selected from various datasets, including COCO [10], ImageNet [11], Clic [12], Inter4K [13], and LOL [1]. Combined with our dimming module, MixHQ allows us to create artificial training pairs for semi-supervised low-light image enhancement.

## 2. Related Work

Low-light image enhancement is a widely recognized task in the field of computer vision, attracting significant attention from researchers. Over the years, various classic approaches have emerged, including techniques such as histogram equalization, gamma correction, and more complex algorithms such as LIME (Low-light Image Enhancement) [14]. These classic methods are known for their remarkable speed and generalizability. However, they often produce images of relatively lower quality compared to more recent deep learning techniques.

Deep learning-based approaches for low-light image enhancement have gained popularity due to their ability to generate visually appealing images. Several methods [1,2,3,4] have achieved notable results in this domain. In RetinexNet [1], the authors proposed to use a neural network for retinex decomposition. The model is trained to reconstruct both reflectance and illumination to match the well-lit images’ maps. KinD and KinD++ [2,3] share a similar idea but use separate modules for each of the maps.

One of the recent approaches, LLFlow [4], utilizes a normalizing flow that learns a distribution of images under normal light conditions given a low-light one. In addition, an encoder is used to extract an illumination-invariant color map that is then injected into the normalizing flow. Authors in [15] propose to adjust the diffusion model for low-light image enhancement. The model utilizes sampling in a pyramid resolution style by progressively increasing resolution in one reverse process. Moreover, this approach uses a global corrector to alleviate the global degradation that may occur in the reverse process. The diffusion-based approach proposed in [16] boosts the performance of low-light enhancement by regularizing the ODE trajectory. Authors in [17] postulate using a wavelet-based conditional diffusion model (WCDM) that leverages the generative power of diffusion models to produce results with satisfactory perceptual fidelity.

In most of the approaches [1,2,3], authors use a UNet [8] convolutional architecture in which the skip connections allow for modeling features of lower frequencies, producing results of higher visual quality. In [5,18], researchers adopt transformer architectures rather than relying solely on convolutional networks. LLNeRF [19] employs the Neural Radiance Fields architecture to improve the visibility of dark scenes by using multiple views of the object.

Most of the previously mentioned approaches rely on datasets consisting of paired images in which one image is captured under low-light conditions, and the other is a corresponding well-lit reference. By learning the mapping from dark to well-lit images, these models can enhance low-light images effectively. However, it is important to note that these paired datasets represent the distribution specific to the cameras used, limiting the generalization of these approaches to other camera models.

Despite the challenges associated with low-light image enhancement, there have been attempts to develop unsupervised approaches using deep learning techniques. For example, EnlightenGAN [6] proposes to leverage a large dataset of unpaired light and dark images and employs adversarial training to enhance low-light images. Another unsupervised method [7] utilizes the observation that the feature maps of trained neural networks are very similar for natural images and for the histogram equalization of the corresponding dark images. Zero-DCE [20] is a zero-shot approach, which formulates low-light image enhancement through monotonic curve estimation with many carefully designed constraints that do not rely on paired or unpaired datasets.

While unsupervised models offer flexibility and the potential to generalize to different cameras, due to the inherent nature of adversarial training, they often fall short in terms of producing high-quality images compared to approaches that employ ground-truth-based loss functions. The lack of explicit supervision in unsupervised methods makes achieving the desired level of image quality and natural color preservation challenging.

LED [21] is a few-shot method for low-light enhancement, which utilizes artificial dark images with synthetic noise to transfer the knowledge of enhancing real dark images. However, this method works only with raw images, whose noise characteristics are specific to sensor errors, while RGB images are post-processed with different pipelines for which we cannot assume any prior knowledge. DRBN [22] aims to make use of both paired and unpaired data by introducing a semi-supervised approach. Initially, it learns to predict a trajectory of corrections through a recursive architecture. Subsequently, a model that combines all corrections into the final prediction is trained in an adversarial manner, eliminating the need for paired images in the second phase. However, the training of the first part of the model still necessitates an extensive paired dataset, given the challenging nature of the task and the complexity of the architecture.

To fill the gap between approaches requiring large paired datasets and fully unsupervised methods, we propose a framework that requires only a few examples with well-fit references to achieve competitive results for the models that require a complete set of pairs for training.

## 3. Our Method

This section provides a detailed description of Dimma, a novel model for enhancing low-light images. Our approach comprises two key components: dimming and brightening modules (see Figure 1). The first module mimics color distortion made by specific cameras to create darkened images. These are further used to train the brightening module represented by the UNet network responsible for restoring the original image.

### 3.1. Dimming Module

**Retinex decomposition**. Previous work [1,2,3] has shown that the retinex decomposition of an image I to illumination L and reflectance R helps models achieve a better quality of restored images. However, most of the works attempt to do this using neural networks, making it slow and introducing reconstruction loss. On top of that, to train such a network, an extensive paired dataset is required. Our approach is based on channel-wise normalization introduced in [4] in which the *i*-th channel of color map R can be calculated as:

(1) $$R_{i}=I_{i}⊘\left(\frac{1}{3}\sum_{i=1}^{3}I_{i}\right),$$

where ⊘ represents Hadamard (element-wise) division, $I_{i}$ and $R_{i}$ represent the *i*-th channel of image I and color map R, respectively. Assuming that the color map is a light-invariant reflectance known from retinex theory and taking into account that the image is a Hadamard product of reflectance and illumination, we obtain the illumination equal L=I⊘R.

**Figure 2:**
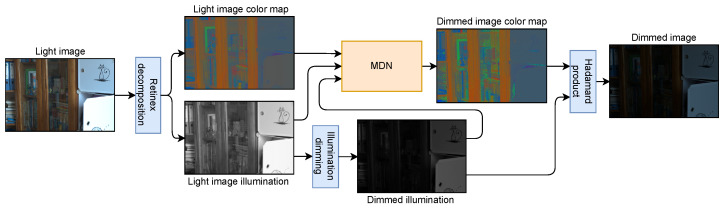
The dimming module first splits the light image into a color map and illumination. The illumination is dimmed using lightness mapping statistics calculated on a few real image pairs. Then, the color map and both illuminations are put into the MDN to sample camera-specific noise which, combined with dimmed illumination, creates the dimmed image. MDN and lightness statistics are calculated from real image pairs; therefore, the dimming process adapts to images taken by the original device, making it ideal for training brightening models on unpaired images.

**Dimming process**. In Figure 2, we present the dimming procedure that creates the corresponding dark images essential for training the brightening module in the second stage. The initial photo is split into illumination L and color map R components using retinex decomposition described in the previous paragraph. Next, illumination dimming is applied on L to create the darker equivalent $L_{D}$. This process involves mean and deviations of mapping between light and dark illumination values calculated from a few training pairs. This process is described in the Appendix A. To align the dimming process to specific camera conditions, we utilize a Mixture Density Network (MDN), which is responsible for adjusting color map R. This module is trained in supervised mode using a few labeled examples, for which the corresponding ground-truth color map is known. The output dark image $I_{D}$ is created by the application of the Hadamard product between dark illumination $L_{D}$ and color map $R_{D}$ sampled from the MDN component $I_{D}=R_{D}⊙L_{D}$.

**Figure 3:**
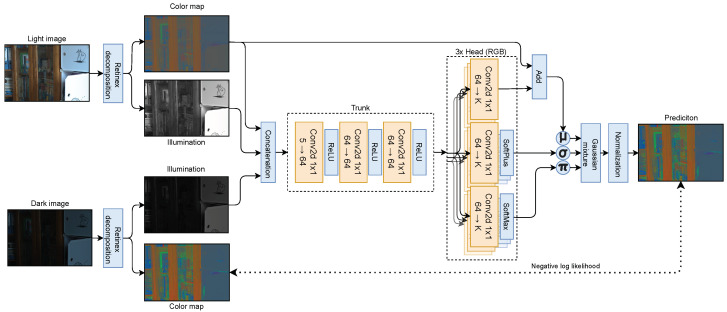
Mixture Density Network (MDN) is the model used in our dimming module for modeling color map distortion of low-light images based on a light image color map and illuminations from both images. The network has a common convolutional trunk and three heads, each for one RGB color. It returns parameters of a mixture of Gaussians from which the dark color map is sampled. Thanks to using only 1 × 1 convolutions and a Gaussian mixture, the model treats each pixel independently and therefore perfectly aligns with the noise modeling task.

**Figure A1:**
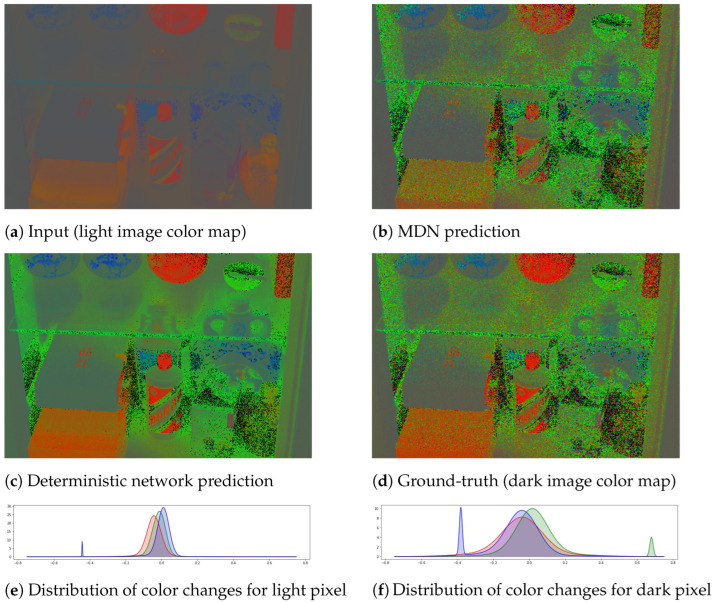
Output of the Mixture Density Network trained on 8 image pairs. (**a**–**d**) show how the dimming module aligns with real image pairs and compare the MDN output with the output of a deterministic convolutional network. We can see that the deterministic approach returns the expected value of each pixel instead of aligning with the true noise distribution. MDN, thanks to its probabilistic nature, better models the dark image noise. (**e**,**f**) illustrate the MDN’s output for gray pixels with different illumination differences (**e**) from 0.2 to 0.15 and (**f**) from 0.2 to 0.05. The characteristic of images from the LOL dataset is that green noise appears in darker regions, which is visible in the (**f**) distribution. The second observation is that the standard deviation is higher for dark images, making them more noisy. We can see that in image (**b**).

**Mixture Density Network (MDN)**. The architecture of the MDN network is provided in Figure 3. This component is responsible for calibrating the color map crucial for creating a darker image. We suggest using a probabilistic model for the following reasons. First, we aim to mimic the noise produced by the camera on the same scene in low-light conditions. The MDN network is capable of modeling complex camera-specific noise, as shown in the Appendix D in Figure A1. Second, we train this component using only a few examples, so the precise color map prediction may be difficult to obtain using limited data. Third, the randomness injected into the process of generating dark images serves as a regularization technique for the brightening module. The ablation study discussed in details in Section 4 shows the rationality of our approach.

The MDN module takes color map R from the light image I and illuminations L and $L_{D}$ from both light and dark images. This enables the network to learn color noise, considering the local brightness of the source and target image. This approach is motivated by the observation that dark regions in photos tend to be more noisy. The color map R and illuminations L and $L_{D}$ are concatenated to create 5-channel input. To make the model as general as possible and to avoid overfitting while training on only a few images, we use only 1 × 1 convolution filters in the MDN architecture. Practically, it means that we process 5-dimensional representations for each pixel independently, with the MLP module sharing the weights across the pixels. Let $x_{i,j}=\left[r_{i,j},l_{i,j},l_{D,i,j}\right]$ denote the 5-dimensional representation that concatenates the pixel values at (i,j) location from input color map R and illuminations L and $L_{D}$, respectively. The distribution over $r_{D,i,j,k}$ representing the scalar element of $R_{D}$ on the (i,j)-th position in channel *k* can be described as:

(2) $$\begin{array}{c} p\left(r_{D,i,j,k}|x_{i,j};\theta \right)=\sum_{m=1}^{M}\pi_{k,m}\left(x_{i,j};\theta \right)\phi_{D,i,j,k,m}, \end{array}$$

where *M* is the number of components in a mixture of Gaussians, θ represents the parameters of the MDN network, and the density of a single Gaussian $\phi_{D,i,j,k,m}$ is defined as:

(3) $$\begin{array}{c} N(r_{i,j,k}+\mu_{k,m}\left(x_{i,j};\theta \right),\sigma_{k,m}\left(x_{i,j};\theta \right)), \end{array}$$

where $\pi_{k,m}\left(x_{i,j};\theta \right)$, $\mu_{k,m}\left(x_{i,j};\theta \right)$, and $\sigma_{k,m}\left(x_{i,j};\theta \right)$ are the parameters of Gaussian mixture predicted by the MDN. Each color channel has its own head, producing means $\mu_{k,m}\left(x_{i,j};\theta \right)$, standard deviations $\sigma_{k,m}\left(x_{i,j};\theta \right)$, and mixing coefficients $\pi_{k,m}\left(x_{i,j};\theta \right)$. The means produced by the model are added to the original color map to make the network predict color differences instead of the total color map. In this way, we make the task easier for the MDN because it was trained on the limited data, which do not contain most of the hues. The training dataset size was also the reason we trained the model to predict color maps instead of entire dark images, which makes it more invariant to illumination conditions in the training data. The joined distribution over $R_{D}$ can be defined as:

(4) $$p\left(R_{D}|R,L,L_{D};\theta \right)=\prod_{i,j,k}p\left(r_{D,i,j,k}|x_{i,j};\theta \right).$$

Assuming access to few (*K*) labeled training examples $D_{K}={\left\{\left(I^{\left(n\right)},I_{D}^{\left(n\right)}\right)\right\}}_{n=1}^{K}={\left\{\left(R^{\left(n\right)},L^{\left(n\right)},R_{D}^{\left(n\right)},L_{D}^{\left(n\right)}\right)\right\}}_{n=1}^{K}$ composed of light $I^{\left(n\right)}$ and corresponding dark images $I_{D}^{\left(n\right)}$, we optimize the θ parameters of the MDN by minimizing the conditional negative log-likelihood:

(5) $$\theta^{*}=\arg\min_{\theta }\sum_{n}\sum_{i,j,k}-\log p(r_{D,i,j,k}^{\left(n\right)}|x_{i,j}^{\left(n\right)};\theta ),$$

where $p(r_{D,i,j,k}^{\left(n\right)}|x_{i,j}^{\left(n\right)};\theta )$ is given by Equation (2). The vector $x_{i,j}^{\left(n\right)}=\left[r_{i,j}^{\left(n\right)},l_{i,j}^{\left(n\right)},l_{D,i,j}^{\left(n\right)}\right]$ concatenates the pixel values at (i,j) location from the *n*-th example color map $R^{\left(n\right)}$ and from illuminations $L^{\left(n\right)}$ and $L_{D}^{\left(n\right)}$.

### 3.2. Brightening Module

**Figure 4:**
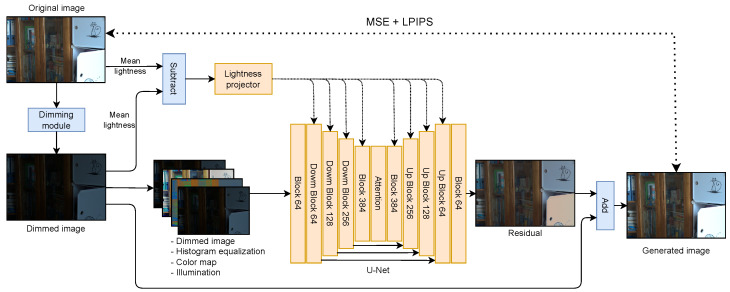
Architecture of our brightening module. In the training process, we used the difference between original and dimmed image lightness to train the model to understand the concept of lightness. Later, in the inference mode, this is provided by the user.

Once the dimming module has been trained, it can be utilized for unsupervised training of a brightening model, as depicted in Figure 1. We chose to use UNet [8], as it proved to be a very good architecture for the low-light image enhancement task [2,3,23]. We decided to further improve it by using conditioning known from diffusion models [24,25,26] to give the model information about the desired target lightness. By injecting lightness into feature maps, the model acquires a lot of information regarding the image’s final appearance. Consequently, it can generate more visually realistic images across various brightness levels. Previous works attempted to achieve this by scaling the image illumination by the desired lightness [2,4,5,15,16,23], which is a more straightforward approach but could not lead to visually appealing images with varying brightness levels that differ from those present in the training set. The detailed architecture of our brightening module is shown in Figure 4.

Let $U_{\beta }$ be a brightening UNet network with parameters β. Inspired by [4], our model takes the concatenation of the original dark image $I_{D}$, its histogram equalization $H(I_{D})$, color map $R_{D}$, and illumination as input $L_{D}$. Compared to reference approaches, our model also takes a lightness degree Δm that controls the light level of the output image. This information is injected into UNet in a similar way as time embedding is used in diffusion models. Thanks to this approach, we can control the level of lightness while enhancing the low-light image. The model returns the residual map U that is further added to the dark image $I_{D}$ to obtain the output image $I=I_{D}+U$. We use sigmoid activation functions on the output of $U_{\beta }$, as this aligns with the intuition that the model should only increase the image’s brightness.

The model $U_{\beta }$ is trained using the set of light images and the set of the few real image pairs $D_{N}\cup D_{K}$, where $D_{N}={\left\{I^{\left(n\right)}\right\}}_{n=1}^{N}={\left\{\left(R^{\left(n\right)},L^{\left(n\right)}\right)\right\}}_{n=1}^{N}$, and where $D_{K}$ contains the few image pairs used to train the dimming module. We choose a pair from $D_{K}$ with the probability *p*; otherwise, we select the light image from $D_{N}$. In this case, we do not have access to illumination $L_{D}$. We solve this issue by calculating $L_{D}=\Phi ⊙L$, where components of dimming matrix Φ are sampled from the normal distribution $N(\gamma \cdot \mu_{l_{i,j}},\alpha \cdot \sigma_{l_{i,j}}^{2})$, where $l_{i,j}$ represent the element in light illumination L. The values of $\mu_{k}$ and $\sigma_{k}$ are means and deviations for each light value calculated from the few training pairs $D_{K}$, where k∈{0,…,255}. The detailed estimation procedure is provided in Appendix A. In addition, we propose to scale the Gaussian means (Equation (2)) by parameter γ, where the values are sampled from the uniform distribution $\gamma ∼U(a_{0},a_{1})$. Thanks to this, we achieve diverse levels of darkness for output images. Second, we utilize the temperature trick usually applied in generative models [27] by scaling the variances from Equation (2) by α<1. The light image $I^{\left(n\right)}$ together with generated dark image ${\tilde{I}}_{D}^{\left(n\right)}$ sampled from the MDN representing the dimming module is further used as one of the training pairs to estimate the parameters β of the brightening component. The model $U_{\beta }$ requires generated dark image ${\tilde{I}}_{D}^{\left(n\right)}$, the corresponding histogram equalization $H({\tilde{I}}_{D}^{\left(n\right)})$, color map ${\tilde{R}}_{D}^{\left(n\right)}$, and illumination as input ${\tilde{L}}_{D}^{\left(n\right)}$. In addition, the parameter $\Delta m^{\left(n\right)}$ that controls the light level should be provided to the model. During training, the $\Delta m^{\left(n\right)}$ is calculated as the difference between the mean lightness of the original light image $I^{\left(n\right)}$ and generated dark image ${\tilde{I}}_{D}^{\left(n\right)}$. In the inference mode, the user provides a value to achieve different levels of light. The total loss used to train the model $U_{\beta }$ is represented by the sum of the mean squared error $L_{MSE}$ and the perceptual loss $L_{P}$:

(6) $$\begin{array}{cc} L_{MSE}+L_{P}= & \sum_{n=1}^{N}\left|\right|I^{\left(n\right)}-\left({\tilde{I}}_{D}^{\left(n\right)}+U^{\left(n\right)}\right){\left|\right|}_{2}^{2}+\\ & \sum_{n=1}^{N}\lambda ||f\left(I^{\left(n\right)}\right)-f\left({\tilde{I}}_{D}^{\left(n\right)}+U^{\left(n\right)}\right){\left|\right|}_{2}^{2}, \end{array}$$

where $U^{\left(n\right)}=U_{\beta }\left({\tilde{I}}_{D}^{\left(n\right)},H\left({\tilde{I}}_{D}^{\left(n\right)}\right),{\tilde{R}}_{D}^{\left(n\right)},{\tilde{L}}_{D}^{\left(n\right)},\Delta m^{\left(n\right)}\right)$ is a residual map returned by UNet for a given dark image ${\tilde{I}}_{D}^{\left(n\right)}$, λ is hyperparameter scaling perceptual loss, and f(·) is the non-trainable feature extractor for perceptual loss. Note that ${\tilde{I}}_{D}^{\left(n\right)}$ represents a different sample from the dimming module for each of the epochs.

**Inference.** After the training process, the dimming module with the MDN is discarded, and only the brightening module is used. Different light settings are controlled using a light condition value from [0,1]. In the validation procedure, we follow previous works [2,3,4,5,15,16,17] and condition the model using the mean pixel value derived from the ground-truth image.

## 4. Experiments

We conducted a series of experiments aiming to demonstrate the superiority of Dimma over previous methods: standard quantitative and qualitative comparisons, a generalizability test, training with limited data, and light-level conditioning. We adopt the experimental setup choices outlined in LLFlow [4]. We compared our model with RetinexNet [1], EnlightenGAN [6], DRBN [22], KinD++ [3], Zero-DCE [20], HEP [7], SNR-Net [18], LLFlow [4], Retinexformer [5], PyDiff [15], and GSAD [16]. For the evaluation of results, we employed PSNR, SSIM [28] on both grayscale and RGB, LPIPS [29,30], and DeltaE and NIQE [31] metrics. We also performed extensive significance tests on the obtained results. Our methodology is described in Appendix C, and the results are presented in Table A2. Finally, we empirically justified our design choices regarding the dimming module and final fine-tuning of Dimma.

**Quantitative**. The first experiment involved a quantitative comparison on the LOL dataset [1]. In the first phase of semi-supervised training, we used 3, 5, or 8 real image pairs from the LOL dataset to train the dimming module. For the second phase (see Figure 1), we introduced the MixHQ dataset, which contained more than 15,000 natural images from COCO2017 [10], ImageNet [11], Clic [12], Inter4K [13], and light images from the train split of the LOL dataset. We selected good-quality images only, using the rules described in Appendix B. Those were used as unlabeled samples for the training of the brightening module. For validation, we used 5 pairs from LOL, and we tested all models on 15 pairs from the LOL test split.

**Figure 5:**
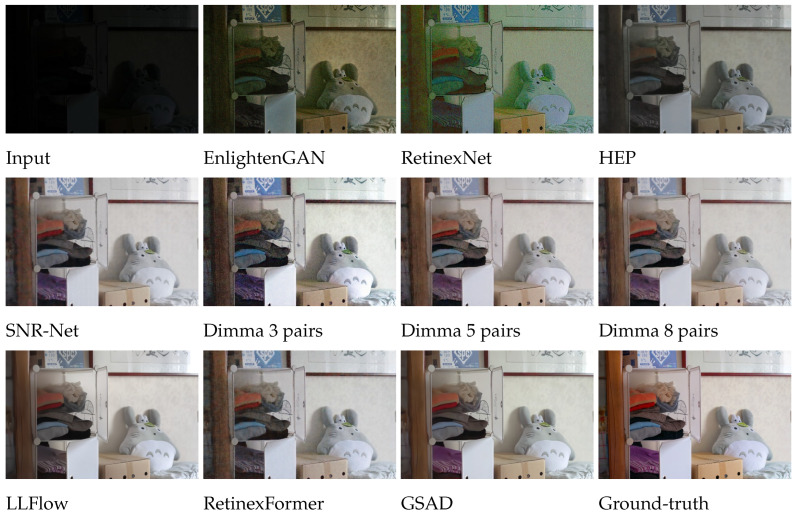
Visual comparison of Dimma with unsupervised and fully-supervised low-light image enhancement methods on the LOL dataset.

As depicted in Table 1, our semi-supervised models trained on a few real image pairs surpass the unsupervised methods by a large margin and closely approach the fully supervised methods that were trained on many image pairs. This introduces a new trade-off between the number of pairs and the quality of generated images. For visual comparison, see Figure 5, Figure A3, Figure A4, Figure A5, Figure A6 and Figure A7 in Appendix E showing the results obtained for various images, including extremely dark or high-contrast ones. To measure the significance of the results, we performed statistical tests using the PSNR metric between HEP [7] and Dimma trained on 3, 5, and 8 pairs, and on LOL [1] and VE-LOL [22]. Our method demonstrates superior performance with a highly significant *p*-value of less than $1\times 10^{-7}$, indicating an extremely low probability of error.

Additionally, we measured the latency of our approach together with a selected group of reference methods. The test was performed on the Kaggle platform with P100 GPU, and we tested all methods compatible with the environment. The results depicted in Table 2 demonstrate the superiority of simple unsupervised methods in latency, with Dimma being a faster alternative compared to more complex supervised approaches.

**Generalizability**. We proceeded with a cross-dataset comparison on the VE-LOL dataset [32], focusing exclusively on models trained on the LOL dataset and unsupervised methods. This particular experiment aimed to evaluate the ability of our method to generalize to unseen image sources. We used 100 real and 100 synthetic pairs from VE-LOL as a test set and evaluated the same methods mentioned in the previous paragraph.

**Table 1:** Quantitative comparisons of the LOL and VE-LOL datasets separated by slashes. All of the models were trained on LOL. The best results are in bold, and the arrows indicate whether the metric should be minimized or maximized. In order to obtain all metrics, we validate each method using code provided on GitHub repositories, and we follow the LLFlow [4] setup. The table is divided into three sections: zero-shot and unsupervised, semi-supervised, and fully-supervised. *—DRBN results were taken from [33] due to problems with running the code provided by the authors.

Method	PSNR ↑	SSIM ↑	RGB-SSIM ↑	LPIPS ↓	DeltaE ↓	NIQE ↓	Train Pairs ↓
Zero-DCE	14.86/16.07	0.65/0.58	0.56/0.47	0.34/0.48	18.82/18.51	8.22/8.92	0
EnlightenGAN	17.48/**16.90**	0.70/0.62	0.65/0.56	0.32/0.43	14.48/16.45	4.89/6.88	0
HEP	**20.23**/16.55	**0.84**/**0.69**	**0.79**/**0.67**	**0.17**/**0.36**	**11.20**/**15.77**	**3.30**/**5.00**	0
DRBN *	15.12/-	0.472/-	-/-	0.316/-	-/-	-/-	689
Dimma 3 pairs	24.44/23.20	0.84/**0.85**	0.78/0.81	0.23/**0.22**	7.91/9.07	3.29/3.66	3
Dimma 5 pairs	24.93/23.25	0.85/**0.85**	0.78/0.81	0.22/**0.22**	7.64/9.30	3.25/3.70	5
Dimma 8 pairs	**25.21**/**23.41**	**0.86**/**0.85**	**0.80**/**0.82**	**0.20**/**0.22**	**7.23**/**8.99**	**3.21**/**3.68**	8
RetinexNet	16.77/14.68	0.52/0.46	0.42/0.36	0.47/0.65	15.89/20.98	9.73/10.36	485
KinD++	21.80/20.41	0.88/0.79	0.83/0.76	0.16/0.28	8.50/11.37	4.00/4.47	460
SNR-Net	24.61/22.93	0.90/0.76	0.84/0.70	0.15/0.32	6.85/11.32	4.02/4.14	485
LLFlow	25.19/22.38	**0.93**/0.73	0.86/0.69	0.11/0.32	6.40/11.13	4.08/5.83	485
Retinexformer	27.18/24.85	0.90/0.87	0.85/0.84	0.13/0.18	5.52/7.74	**2.95**/**3.62**	485
PyDiff	27.17/**27.07**	**0.93**/**0.91**	**0.88**/**0.88**	0.10/**0.15**	**5.33**/**6.98**	4.01/4.46	485
GSAD	**27.69**/24.85	0.92/0.87	**0.88**/0.85	**0.09**/**0.15**	5.36/7.78	4.14/4.92	485

Results depicted in Table 1 show the ability to preserve good quality in a cross-dataset scenario. Specifically, we observe that Dimma has a much smaller quality drop than other methods. For example, HEP [7], while obtaining astonishing results on the in-domain dataset, failed to achieve good results in the cross-domain setup.

**Limited data**. To examine the usefulness of our framework in real scenarios when we have only a few real image pairs, we collected a dataset that we called FewShot-Dark (FS-Dark). The collecting procedure was similar to LOL [1]—we took two photos of the same scene with different camera light exposures. FS-Dark consists of a total of 14 image pairs (6 training, 4 validation, and 4 testing) taken by a Samsung Galaxy M52 mobile phone. For a fair comparison, we trained our Dimma model, LLFlow [4], and SNR-Net [18]. We also validated the LLFlow trained on the LOL dataset.

**Figure 6:**
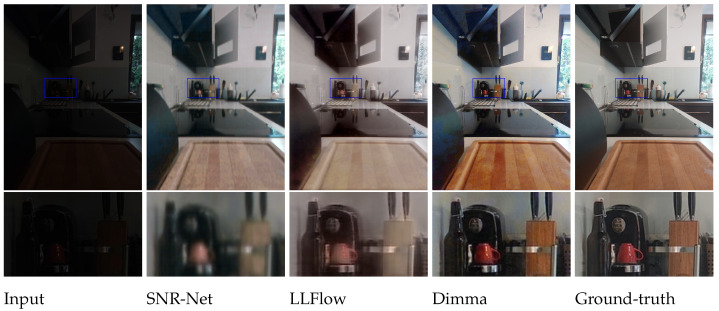
Results for the FewShot-Dark dataset. All models were trained on the same training set consisting of six training image pairs. The training setup for each method was the same as for the LOL dataset.

**Figure A2:**
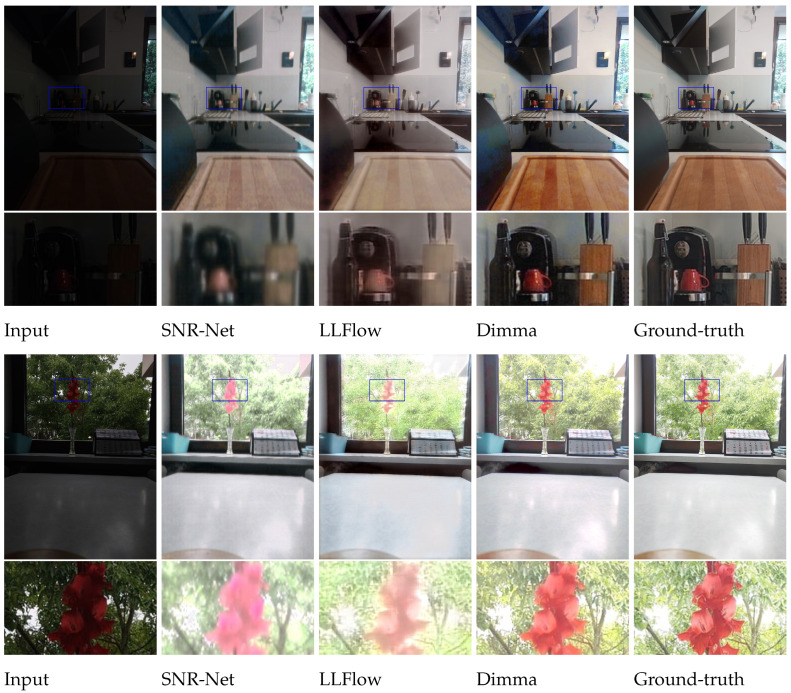

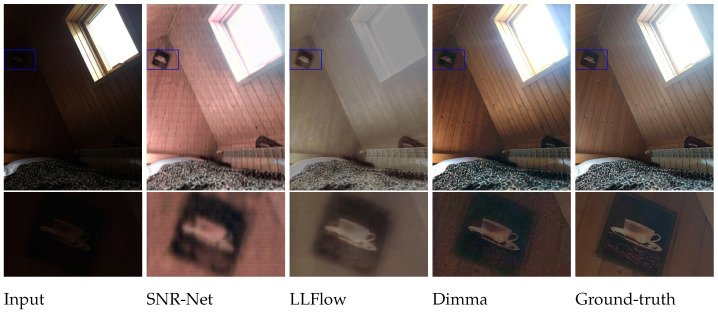
Results on three test image pairs from our FewShot-Dark dataset. All three methods were trained on the training split of this dataset consisting of only six training image pairs. The training setup for each method was the same as for the LOL dataset.

**Figure A3:**
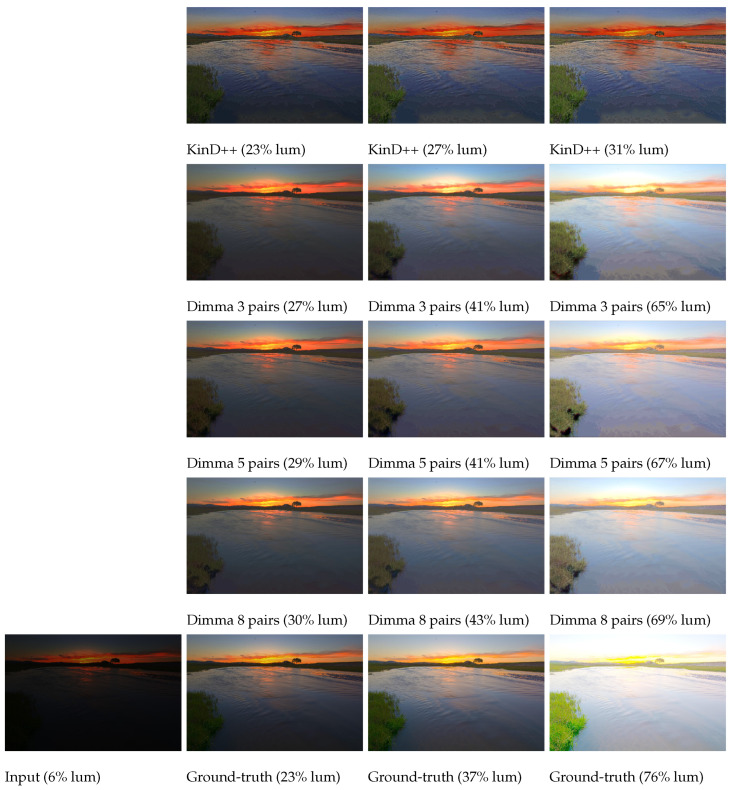
Visualization of low-light enhancement with different brightening factors for Dimma and KinD++ on the image from the SICE dataset. Both models were conditioned by the illumination values of the ground-truth images displayed at the bottom. The average illumination of each image is shown in brackets, indicating the accuracy of the models in generating images with specific light levels.

**Figure A4:**
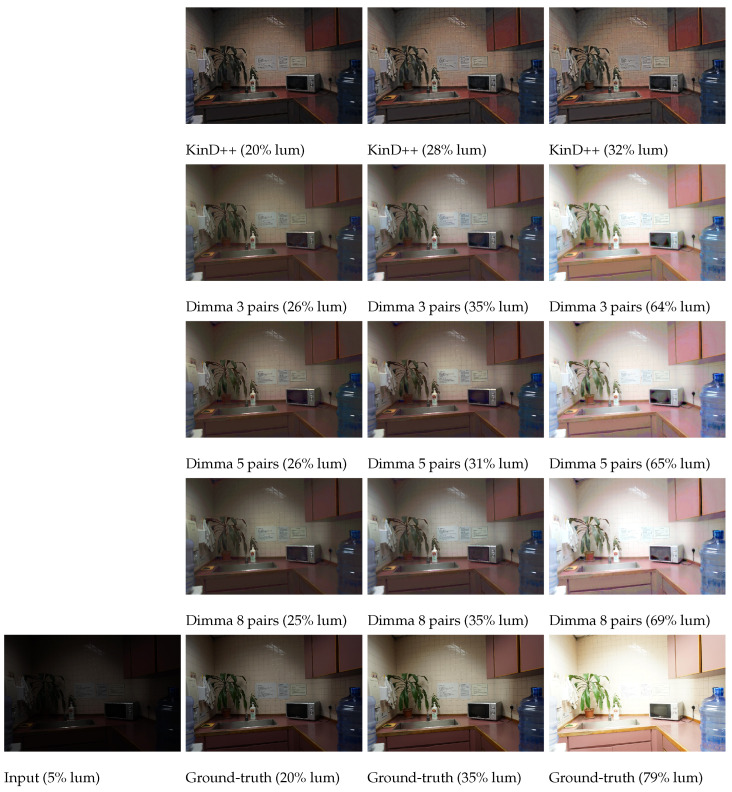
Visualization of low-light enhancement with different brightening factors for Dimma and KinD++ on the images from the SICE dataset. Both models were conditioned by the illumination values of the ground-truth images displayed at the bottom. The average illumination of each image is shown in brackets, indicating the accuracy of the models in generating images with specific light levels.

**Figure A5:**
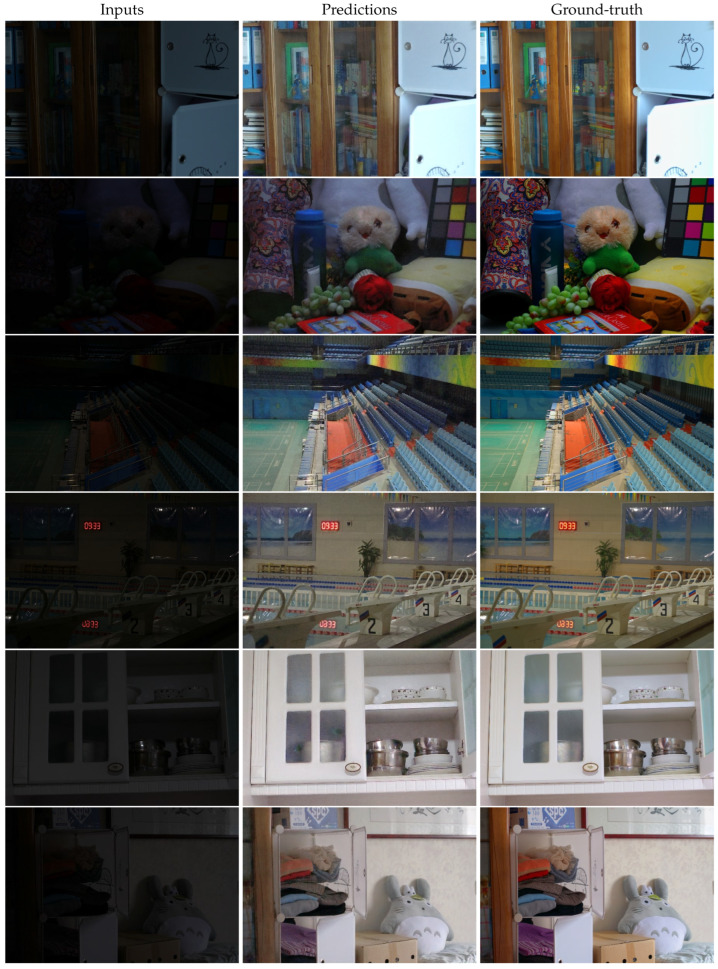
Visual results of the Dimma model trained on 3 pairs from the LOL dataset.

**Figure A6:**
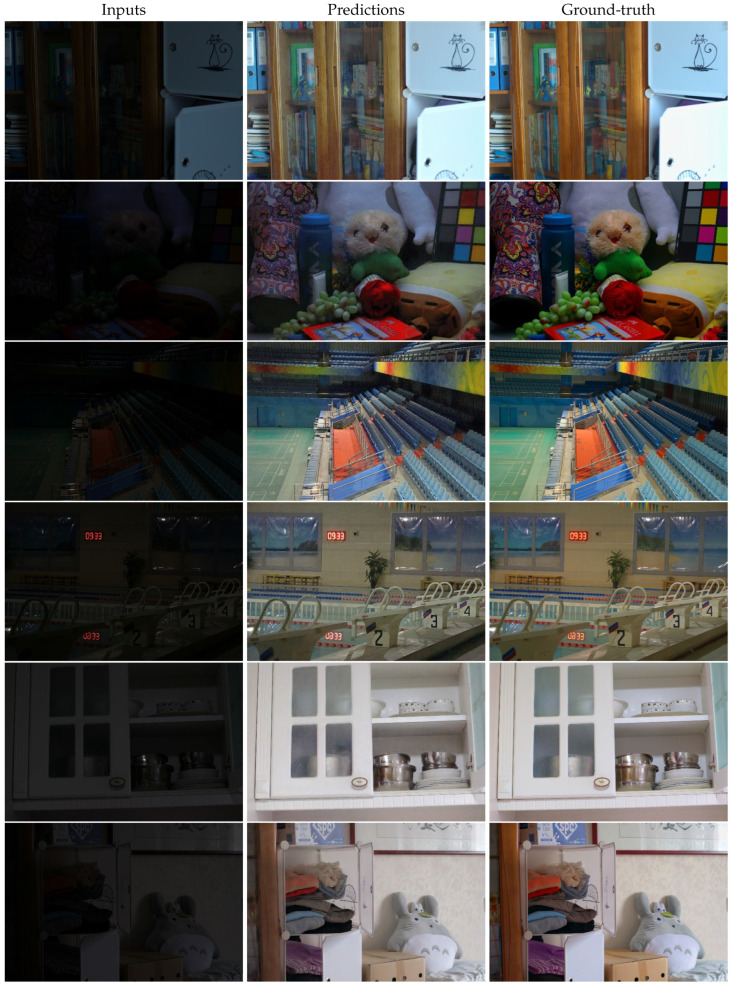
Visual results of the Dimma model trained on 5 pairs from the LOL dataset.

**Figure A7:**
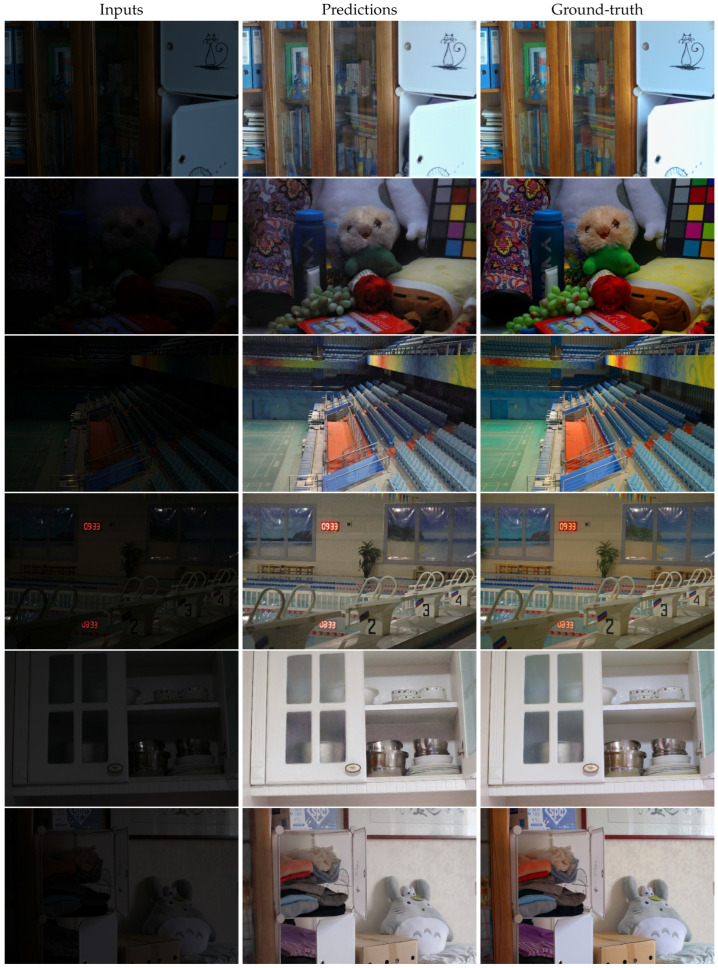
Visual results of the Dimma model trained on 8 pairs from the LOL dataset.

The results are shown in Table 3, and the visual comparison is presented in Figure 6. For more image examples, see Figure A2 in Appendix E. Compared to models trained on FS-Dark, our method achieves better results in terms of all metrics, which proves Dimma strongly outperforms other methods when only a few ground-truth pairs are available for training. Dimma also surpasses LLFlow trained on the LOL dataset in almost all metrics, which suggests that models trained on one data collection might not generalize well to the others.

**Table 2:** Runtime analysis of VE-LOL for selected methods performed in the Kaggle P100 environment. We measure average processing time of a single image in seconds.

Method	Time [s]
LLFlow	0.438 ± 0.168
SNR-Net	0.065 ± 0.004
EnlightenGAN	0.028 ± 0.041
Dimma (ours)	0.026 ± 0.051
HEP	0.009 ± 0.046
RUAS	0.009 ± 0.055
Zero-DCE	0.004 ± 0.044

**Light conditioning**. To compare our light conditioning mechanism, we used the SICE [34] dataset consisting of image sequences with different lightness. Since most low-light image enhancement methods (e.g., [2,3,4,23]) are capable of lightness conditioning, we created the visual comparison of generated images with different conditioning values. Thanks to the lightening conditioning mechanism of UNet, we achieved remarkable precision in generating images with a certain level of lightening. The qualitative results, placed in Appendix E, emphasize the superiority of Dimma in terms of light conditioning.

**Ablation study**. To further establish the efficacy of our approach, we compared the results of semi-supervised training using Dimma with training UNet without the dimming module. The intention behind these experiments was to validate the rationality of our dimming module with MDN on the extremely small dataset. Using the dimming module exposes UNet to more natural images than training only on paired ones, which increases the model’s knowledge of the natural-light photos’ distribution. However, this comes at the cost of using synthetic inputs, which may not perfectly represent the true distribution of dark images. We also showed that using a deterministic convolutional network with the same architecture as the backbone of the MDN does not produce results as appealing as our MDN-based dimming module does.

Our ablation study examines the usefulness of the learnable dimming module. We compare it to a more naive approach of training only the UNet on the very same few pairs. Table 4 shows the rationality of the Dimma pipeline. In Figure A1 in Appendix D, we show the visual comparison of the color map generated by the MDN and the deterministic neural network. The comparison shows that the deterministic approach always generates the expected value of the color map pixels and cannot synthesize any variation due to the lack of stochasticity. The MDN, on the other hand, employs a mixture of Gaussians, which introduces randomness and creates distorted color maps that better align with the color maps of real-world dark images.

To further study our semi-supervised setup, we examined different probabilities *p* of choosing the real image pair during brightening module training. Higher *p* values move our model closer to the standard supervised method, which we proved to be less effective in the previous ablations. On the other hand, Dimma with small *p* values relies strongly on the data generated by the MDN and therefore can overfit to synthetic pairs. We examined three different values of *p*, and the results are shown in Table 5. Based on this study, we chose the value of p=0.75.

## 5. Conclusions

In this paper, we propose a novel semi-supervised learning approach for low-light image enhancement. We address the problem of an insufficient number of large datasets containing image pairs for various cameras by utilizing only a few real image pairs, which can be easily captured and prepared. Based on these pairs, our model aims to mimic the characteristics of a specific camera in low-light conditions observed in the data. With this approach, we can effectively train existing models to restore dark images captured by that particular camera. Furthermore, our approach allows for the grading of the dimming factor during training, enabling the model to adapt to different light conditions and generate images with any desired illumination. As a result, our method significantly outperforms existing unsupervised models and achieves competitive results compared to fully supervised approaches.

For the FS-Dark dataset with limited training pairs, Dimma is superior to supervised methods [4,18]. This demonstrates Dimma’s ability to make better use of limited training samples. Additionally, Dimma significantly outperforms LLFlow [4] trained on the full LOL dataset in PSNR and DeltaE metrics, and it performs comparably in other metrics. This suggests that large, general, low-light image enhancement datasets are insufficient for different domains, such as those taken by different cameras or having different photo characteristics, highlighting many potential practical uses for Dimma.

**Limitations**. The main limitation of Dimma is the need to train the UNet backbone using a dimming module calibrated for particular camera settings. As a result, training must start from scratch for each new dataset. Secondly, our method achieves good results using only a few paired images, which makes the data-gathering process easier and cheaper but still necessary. Dimma cannot work without paired photos, which are much easier to collect.

As a potential future work, having a more general backbone and changing camera-specific modules on top for different datasets would enable few-shot fine-tuning, limiting the time and resources needed. The second drawback of Dimma highlights that the development of fully unsupervised methods that are on par with supervised models is the next crucial step in low-light image enhancement. A potential improvement in this direction could be replacing the MDN model within the dimming module with conditional GAN models for reflectance distortion, making the training process fully unsupervised, similar to [6].

## Figures and Tables

**Table 3:** Quantitative comparison for FS-Dark. The best results are in bold, and the arrows indicate whether the metric should be minimized or maximized. All of the models were trained on FS-Dark (except for LLFlow (LOL), which was trained on LOL), consisting of as few as 6 training pairs, with the same setup as for LOL (except for the smaller batch size due to the dataset size).

Method	PSNR ↑	SSIM ↑	RGB-SSIM ↑	LPIPS ↓	DeltaE ↓	NIQE ↓
SNR-Net	19.43	0.78	0.75	0.42	9.59	4.61
LLFlow	19.46	0.81	0.79	0.35	9.69	3.50
LLFlow (LOL)	20.77	0.87	**0.86**	**0.19**	7.91	2.74
Dimma (ours)	**25.56**	**0.88**	**0.86**	0.22	**5.77**	**2.67**

**Table 4:** Ablation study on the LOL dataset. The best results are in bold. Each experiment was conducted three times with different training samples. The files that were used for training are listed in Table A1 in Appendix B.

# Pairs	3	5	8
Supervised (w/o dimming module)			
PSNR	21.05 ± 4.16	21.62 ± 4.19	22.18 ± 4.40
SSIM	0.81 ± 0.09	0.82 ± 0.09	0.83 ± 0.09
LPIPS	0.27 ± 0.09	0.25 ± 0.09	0.23 ± 0.08
Semi-supervised with deterministic dimming			
PSNR	22.93 ± 2.31	24.07 ± 2.41	24.26 ± 2.40
SSIM	0.78 ± 0.06	0.78 ± 0.06	0.79 ± 0.06
LPIPS	0.32 ± 0.08	0.31 ± 0.08	0.30 ± 0.08
Semi-supervised with the MDN			
PSNR	23.24 ± 2.25	23.83 ± 2.26	24.47 ± 2.22
SSIM	0.78 ± 0.06	0.79 ± 0.06	0.79 ± 0.05
LPIPS	0.31 ± 0.08	0.30 ± 0.08	0.29 ± 0.07
Semi-supervised with the MDN & few real pairs (Dimma)			
PSNR	**24.44 ± 0.76**	**24.93 ± 0.80**	**25.21 ± 0.36**
SSIM	**0.84 ± 0.01**	**0.85 ± 0.01**	**0.86 ± 0.01**
LPIPS	**0.23 ± 0.02**	**0.22 ± 0.02**	**0.20 ± 0.01**

**Table 5:** Ablation studies on the probability of passing the real image pair instead of the synthetic pair produced by the dimming module. The real pairs are the same as those that were used to train the dimming module. Experiments were conducted on the LOL dataset. We used PSNR and DeltaE as metrics, as they vary the most for different values of *p*. The best results are in bold, and the arrows indicate whether the metric should be minimized or maximized.

# Pairs	*p*	PSNR ↑	DeltaE ↓
	0.50	24.26	9.22
3 pairs	0.75	**24.44**	**7.91**
	0.90	24.21	8.00
	0.50	**24.95**	9.19
5 pairs	0.75	24.93	**7.64**
	0.90	24.85	7.67
	0.50	25.20	8.95
8 pairs	0.75	**25.21**	**7.23**
	0.90	25.13	7.27

## Data Availability

The original data presented in the study are openly available at https://drive.google.com/drive/folders/1wV4MdG8sSFCkRUmwiyPG43Si5Sq0-VzQ?usp=drive_link.

## Appendix A. Detailed Architecture

**Dimming module**. Our dimming module utilizes retinex decomposition to deal separately with the color map and illumination. To manipulate the color map, we use the Mixture Density Network (MDN) to be able to align well with the color distortion distribution present in the dark images. We set the number of mixture components to 8, and each pixel can have its mean and standard deviation sampled from different components. This approach, together with using only convolution with 1 × 1 filters, allows us to treat each pixel as an individual sample, preventing quick overfitting with an insufficient number of pairs. We train the MDN model for 25,000 steps with a starting learning rate set to $1\times 10^{-3}$, with cosine annealing stops at $1\times 10^{-5}$.

Since illumination has only one value per pixel, it is less complicated, and we can model its dynamic without parameterized models. We compute dimmed illumination from the formula:

(A1) $$L_{D}=\Phi ⊙L,$$

where elements of Φ are sampled from the Gaussian distribution:

(A2) $$\phi_{i,j}=N\left(\gamma \cdot \mu_{l_{i,j}},\alpha \cdot \sigma_{l_{i,j}}\right),$$

where γ denotes the dimming factor, α is a noise temperature, and $\mu_{l_{i,j}}$ and $\sigma_{l_{i,j}}$ are means and standard deviations of lightness ratios for each illumination value calculated from the few training pairs:

(A3) $$\begin{array}{cc} \mu_{k} & =\frac{1}{\sum_{n,i,j}I\left(l_{n,i,j}=k\right)}\sum_{n,i,j}\frac{l_{D,n,i,j}I\left(l_{n,i,j}=k\right)}{l_{n,i,j}} \end{array}$$

(A4) $$\begin{array}{cc} \sigma_{k} & =\sqrt{\frac{\sum_{n,i,j}{\left(\frac{l_{D,n,i,j}I\left(l_{n,i,j}=k\right)}{l_{n,i,j}}-\mu_{k}\right)}^{2}}{\sum_{n,i,j}I\left(l_{n,i,j}=k\right)-1}}, \end{array}$$

where k∈(0,255) are all possible light values, $l_{n,i,j}$ is an illumination value from an *n*-th light image at the (i,j) pixel location, and $l_{D,n,i,j}$ is the illumination value of the corresponding pixel from a dark image. Operator I returns 1 if arguments are true and 0 otherwise. In the rare case when $\sum_{n,i,j}I\left(l_{n,i,j}=k\right)$ is 0 or 1, we interpolate values from the closest known lightness values.

Intuitively, for each pixel value from the normally exposed images, we calculate the distribution of its values in the low-light conditions.

**Conditioned UNet**. Our UNet model consists of three upsample, three downsample, and four normal layers. Each layer has two ResNet blocks [26]. We applied an attention mechanism in the bottleneck with a head dimension set to 64. We conditioned UNet layers with a mean lightness difference between the original image and the image we wanted to produce. This difference is projected using standard positional encoding with a sinus function. The output is transformed with a two-layer MLP and injected into the UNet feature map during its forward pass. The implementation of the UNet network is from diffusers [26].

**Training settings**. For the semi-supervised approach, we utilized 3, 5, or 8 pairs from LOL. Each training was repeated three times using different training sets. We trained the UNet on random crops of size 256 × 256, with a batch size of 4. The initial learning rate was set to $10^{-5}$, and we employed a cosine annealing scheduler for 15,000 iterations, with early stopping based on the validation set. The optimization was performed using the Adam optimizer.

Dimming factor $\gamma ∼U(a_{0},a_{1})$ where $a_{0}=0.3$ and $a_{1}=2$, whereas γ<1 produces slightly darker images than those from the training set, and γ>1 creates lighter images. In this way, the model can be trained on a wider range of light conditions.

During the training, there is p=0.75 chance that the model obtains a real image pair and 1−p=0.25 that the artificial dark image will be passed. The real pairs are the same that were used in the dimming module training, so no additional image pairs are required. We conducted an ablation study on the *p* value shown in Table 5.

## Appendix B. Datasets

**MixHQ**. To train UNet in an unsupervised way, we combine five real image datasets to give the model knowledge about the distribution of good-quality photos. During the training process, we dim them according to the Dimma paradigm. The unsupervised training proved to increase the quality of generated images, especially if we do not have too many real image pairs. To provide only the best quality images, we select them based on the following conditions:

- COCO2017 unlabeled—Images with at least a 500 × 500 resolution.
- Clic—Images reduced to 512 × 512 in size.
- ImageNet1K—Images from validation and test sets with resolution between 550 × 550 and 1100 × 1100. We discarded images with white backgrounds.
- Inter4K—The first and the last frame of each clip resized by a factor of 0.3.
- LOL—Original 485 training split. Note that we only used light images without knowledge of the dark image distribution.

**Table A1:** Files from the LOL dataset [1] that are used for semi-supervised training. We train 9 MDN models and 9 UNets to compare the effectiveness of our method. Results are shown in Table 4.

Exp.	# Pairs	File 1	File 2	File 3	File 4	File 5	File 6	File 7	File 8
	3	2.png	5.png	6.png					
1	5	2.png	5.png	6.png	9.png	10.png			
	8	2.png	5.png	6.png	9.png	10.png	12.png	13.png	14.png
	3	17.png	18.png	21.png					
2	5	17.png	18.png	21.png	24.png	25.png			
	8	17.png	18.png	21.png	24.png	25.png	26.png	27.png	28.png
	3	36.png	38.png	39.png					
3	5	36.png	38.png	39.png	40.png	42.png			
	8	36.png	38.png	39.png	40.png	42.png	43.png	44.png	46.png

**Semi-supervised training**. We use different subsets of the LOL training set for semi-supervised learning experiments. Table A1 shows the filenames used in each experiment. Files from this table were used for MDN training, illumination dynamic estimation, and fine-tuning the UNet model after training it on MixHQ in an unsupervised manner.

## Appendix C. Significance Tests

To determine which results show a significant difference compared to existing methods, we conducted a Wilcoxon signed-rank test. This non-parametric test was chosen because each experiment was repeated only three times, making the Wilcoxon test more appropriate for our small sample size. The null hypothesis assumed that the differences between the metrics obtained from Dimma and other methods are centered around 0. The alternative hypothesis suggested that the differences are not centered around 0, indicating that one method outperforms the other. Since we trained three models for each specific number of training pairs, we extended the series threefold to obtain more accurate *p*-values.

The tests were conducted on the LOL [1] and VE-LOL [22] datasets, comparing Dimma models with all other methods. The results are shown in Table A2. To indicate which model is significantly better, we use green to highlight cases where Dimma outperforms the comparison method and red where Dimma performs significantly worse. If the results are not statistically significant, the *p*-values are shown in gray. We set the significance level at 0.01. The table is divided into three sections based on the number of training pairs. Within these sections, we further separate the results into unsupervised, semi-supervised, and supervised methods. We also compare Dimma with itself across different numbers of training pairs to assess the impact of training set size on performance. The lengths of the series used in the test were 45 for LOL and 600 for VE-LOL.

**Table A2:** Statistical significance of the differences between Dimma trained on 3, 5, and 8 pairs and other low-light image enhancement methods. Test were performed on the LOL and VE-LOL datasets, and the results are separated by slashes. The *p*-values were obtained using the Wilcoxon signed-rank test. The *p*-values are colored in green if Dimma is significantly better, red if Dimma is significantly worse, and gray if there is no significant difference. We chose the significance level of 0.01.

**Dimma 3 Pairs**						
Methods	PSNR	SSIM	RGB-SSIM	LPIPS	DeltaE	NIQE
Zero-DCE	<0.01/<0.01	<0.01/<0.01	<0.01/<0.01	<0.01/<0.01	<0.01/<0.01	<0.01/<0.01
EnlightenGAN	<0.01/<0.01	<0.01/<0.01	<0.01/<0.01	<0.01/<0.01	<0.01/<0.01	<0.01/<0.01
HEP	<0.01/<0.01	0.126/<0.01	<0.01/<0.01	<0.01/<0.01	<0.01/<0.01	0.823/<0.01
Dimma 5 pairs	0.017/0.066	0.763/0.259	<0.01/<0.01	<0.01/0.069	0.150/<0.01	0.225/0.062
Dimma 8 pairs	<0.01/<0.01	<0.01/<0.01	<0.01/<0.01	<0.01/<0.01	<0.01/0.280	0.072/0.396
RetinexNet	<0.01/<0.01	<0.01/<0.01	<0.01/<0.01	<0.01/<0.01	<0.01/<0.01	<0.01/<0.01
KinD++	<0.01/<0.01	<0.01/<0.01	<0.01/<0.01	<0.01/<0.01	0.225/<0.01	<0.01/<0.01
SNR-Net	0.553/0.508	<0.01/<0.01	<0.01/<0.01	<0.01/<0.01	0.014/<0.01	<0.01/<0.01
LLFlow	0.028/<0.01	<0.01/<0.01	<0.01/<0.01	<0.01/<0.01	<0.01/<0.01	<0.01/<0.01
Retinexformer	<0.01/<0.01	<0.01/<0.01	<0.01/<0.01	<0.01/<0.01	<0.01/<0.01	<0.01/0.746
PyDiff	<0.01/<0.01	<0.01/<0.01	<0.01/<0.01	<0.01/<0.01	<0.01/<0.01	<0.01/<0.01
GSAD	<0.01/<0.01	<0.01/<0.01	<0.01/<0.01	<0.01/<0.01	<0.01/<0.01	<0.01/<0.01
**Dimma 5 Pairs**						
Methods	PSNR	SSIM	RGB-SSIM	LPIPS	DeltaE	NIQE
Zero-DCE	<0.01/<0.01	<0.01/<0.01	<0.01/<0.01	<0.01/<0.01	<0.01/<0.01	<0.01/<0.01
EnlightenGAN	<0.01/<0.01	<0.01/<0.01	<0.01/<0.01	<0.01/<0.01	<0.01/<0.01	<0.01/<0.01
HEP	<0.01/<0.01	0.134/<0.01	0.016/<0.01	<0.01/<0.01	<0.01/<0.01	0.671/<0.01
Dimma 3 pairs	0.017/0.066	0.763/0.259	<0.01/<0.01	<0.01/0.069	0.150/<0.01	0.225/0.062
Dimma 8 pairs	0.225/<0.01	<0.01/0.011	<0.01/<0.01	<0.01/<0.01	<0.01/<0.01	0.072/<0.01
RetinexNet	<0.01/<0.01	<0.01/<0.01	<0.01/<0.01	<0.01/<0.01	<0.01/<0.01	<0.01/<0.01
KinD++	<0.01/<0.01	<0.01/<0.01	<0.01/<0.01	<0.01/<0.01	0.107/<0.01	<0.01/<0.01
SNR-Net	0.929/0.258	<0.01/<0.01	<0.01/<0.01	<0.01/<0.01	0.061/<0.01	<0.01/<0.01
LLFlow	0.538/<0.01	<0.01/<0.01	<0.01/<0.01	<0.01/<0.01	<0.01/<0.01	<0.01/<0.01
Retinexformer	<0.01/<0.01	<0.01/<0.01	<0.01/<0.01	<0.01/<0.01	<0.01/<0.01	<0.01/0.123
PyDiff	<0.01/<0.01	<0.01/<0.01	<0.01/<0.01	<0.01/<0.01	<0.01/<0.01	<0.01/<0.01
GSAD	<0.01/<0.01	<0.01/<0.01	<0.01/<0.01	<0.01/<0.01	<0.01/<0.01	<0.01/<0.01
**Dimma 8 Pairs**						
Methods	PSNR	SSIM	RGB-SSIM	LPIPS	DeltaE	NIQE
Zero-DCE	<0.01/<0.01	<0.01/<0.01	<0.01/<0.01	<0.01/<0.01	<0.01/<0.01	<0.01/<0.01
EnlightenGAN	<0.01/<0.01	<0.01/<0.01	<0.01/<0.01	<0.01/<0.01	<0.01/<0.01	<0.01/<0.01
HEP	<0.01/<0.01	0.346/<0.01	0.414/<0.01	<0.01/<0.01	<0.01/<0.01	0.401/<0.01
Dimma 3 pairs	<0.01/<0.01	<0.01/<0.01	<0.01/<0.01	<0.01/<0.01	<0.01/0.280	0.072/0.396
Dimma 5 pairs	0.225/<0.01	<0.01/0.011	<0.01/<0.01	<0.01/<0.01	<0.01/<0.01	0.072/<0.01
RetinexNet	<0.01/<0.01	<0.01/<0.01	<0.01/<0.01	<0.01/<0.01	<0.01/<0.01	<0.01/<0.01
KinD++	<0.01/<0.01	<0.01/<0.01	<0.01/<0.01	<0.01/<0.01	<0.01/<0.01	<0.01/<0.01
SNR-Net	0.712/0.090	<0.01/<0.01	<0.01/<0.01	<0.01/<0.01	0.160/<0.01	<0.01/<0.01
LLFlow	0.884/<0.01	<0.01/<0.01	<0.01/<0.01	<0.01/<0.01	<0.01/<0.01	<0.01/<0.01
Retinexformer	<0.01/<0.01	<0.01/<0.01	<0.01/<0.01	<0.01/<0.01	<0.01/<0.01	<0.01/0.302
PyDiff	<0.01/<0.01	<0.01/<0.01	<0.01/<0.01	<0.01/<0.01	<0.01/<0.01	<0.01/<0.01
GSAD	<0.01/<0.01	<0.01/<0.01	<0.01/<0.01	<0.01/<0.01	<0.01/<0.01	<0.01/<0.01

Overall, Dimma significantly outperforms unsupervised methods across most metrics and even some supervised methods. However, there are cases where certain supervised methods perform significantly better, highlighting that Dimma effectively balances the trade-off between small dataset size and strong performance.

## Appendix D. Ablation Studies

To examine the rationality of our approach, we conducted a series of experiments comparing the supervised approach, standard Dimma method, and Dimma with a deterministic convolutional network as a dimming model. We utilized the same architecture of UNet and the training sets mentioned in Table A1 for all three approaches. The results, presented in Table 4, provide insights into the performance of each method. Our Dimma method consistently outperforms any other approach, especially when the available real image pairs are limited.

Moreover, we aimed to demonstrate the advantages of incorporating a generative model, specifically the Mixture Density Network (MDN), in the dimming procedure. Figure A1 visualizes the output of our dimming module, showcasing its ability to produce noise that closely resembles the real noise found in dark images. By leveraging a distribution-based approach, the MDN generates varying degrees of noise, capturing the nuances of dim photos. Table 4 provides further evidence supporting the superiority of the MDN over the deterministic network. In almost all cases, the results obtained using the MDN consistently surpass or at least match the performance of the deterministic network.

Overall, our experiment demonstrates that the Dimma method, utilizing a generative model like the MDN, yields superior results compared to both standard supervised training and deterministic approaches.

## Appendix E. Visualizations

In this section, we present visual results of our method on different datasets as well as visual comparisons with previous methodologies.

Figure A2 presents the results of Dimma, SNR-Net, and LLFlow on our dataset called FewShow-Dark (FS-Dark). These methods were trained using six pairs of images from FS-Dark’s training set. While SNR-Net and LLFlow perform very well on the larger LOL dataset with many training pairs, they struggle when given only a few training samples. In contrast, Dimma adapts to images taken with different camera setups after just a few training pairs, generating high-quality samples for the FS-Dark dataset.

Figure A3 and Figure A4 demonstrate Dimma’s remarkable ability to produce images with various desired levels of brightness. Each model was guided by the illumination of the ground-truth images presented at the bottom of the figures, aiming to reproduce the distinct brightness levels from the corresponding dark inputs. Dimma excels in generating images that closely match the exact brightness of the ground-truth images. Moreover, the model shows its ability to generate convincingly over-exposed images.

Figure A5, Figure A6 and Figure A7 present the results for the LOL dataset produced by our four models trained on, respectively, 3, 5, and 8 image pairs.
